# Development of an *Ex Vivo*, Beating Heart Model for CT Myocardial Perfusion

**DOI:** 10.1155/2015/412716

**Published:** 2015-06-21

**Authors:** Gert Jan Pelgrim, Marco Das, Ulrike Haberland, Cees Slump, Astri Handayani, Sjoerd van Tuijl, Marco Stijnen, Ernst Klotz, Matthijs Oudkerk, Joachim E. Wildberger, Rozemarijn Vliegenthart

**Affiliations:** ^1^University of Groningen, University Medical Center Groningen, Center for Medical Imaging-North East Netherlands, Department of Radiology, Hanzeplein 1, 9713 GZ Groningen, Netherlands; ^2^Department of Radiology and Cardiovascular Research Institute Maastricht (CARIM), Maastricht University Medical Center, Postbus 5800, 6202 AZ Maastricht, Netherlands; ^3^Siemens AG Healthcare, Forchheim, Germany; ^4^University of Twente, Drienerlolaan 5, 7522 NB Enschede, Netherlands; ^5^LifeTec Group BV, Den Dolech 2, 5612 AZ Eindhoven, Netherlands; ^6^University of Groningen, University Medical Center Groningen, Center for Medical Imaging-North East Netherlands, Hanzeplein 1, 9713 GZ Groningen, Netherlands

## Abstract

*Objective*. To test the feasibility of a CT-compatible, *ex vivo*, perfused porcine heart model for myocardial perfusion CT imaging. *Methods*. One porcine heart was perfused according to Langendorff. Dynamic perfusion scanning was performed with a second-generation dual source CT scanner. Circulatory parameters like blood flow, aortic pressure, and heart rate were monitored throughout the experiment. Stenosis was induced in the circumflex artery, controlled by a fractional flow reserve (FFR) pressure wire. CT-derived myocardial perfusion parameters were analysed at FFR of 1 to 0.10/0.0. *Results*. CT images did not show major artefacts due to interference of the model setup. The pacemaker-induced heart rhythm was generally stable at 70 beats per minute. During most of the experiment, blood flow was 0.9–1.0 L/min, and arterial pressure varied between 80 and 95 mm/Hg. Blood flow decreased and arterial pressure increased by approximately 10% after inducing a stenosis with FFR ≤ 0.50. Dynamic perfusion scanning was possible across the range of stenosis grades. Perfusion parameters of circumflex-perfused myocardial segments were affected at increasing stenosis grades. *Conclusion*. An adapted Langendorff porcine heart model is feasible in a CT environment. This model provides control over physiological parameters and may allow in-depth validation of quantitative CT perfusion techniques.

## 1. Introduction

Computed tomography (CT) has become the premier noninvasive imaging modality for the noninvasive evaluation of the coronary arteries. For the functional assessment of coronary artery disease (CAD), the sole diagnosis of coronary luminal narrowing is often limited, especially in case of 30–70 percent (intermediate) grade stenosis [1]. Usually, additional testing on the impact of stenosis on myocardial perfusion is needed. To date, CT is not commonly used in daily clinical practice worldwide, except for several leading clinics in CT imaging. However, recent evidence suggests that state-of-the-art CT scanners allow evaluation of myocardial blood supply, on top of the interrogation of coronary morphology [2]. This includes quantification of myocardial perfusion using dynamic perfusion techniques in second-generation dual source CT (DSCT) scanning [3, 4]. Measurement of absolute myocardial perfusion can enhance the diagnostic accuracy for hemodynamically significant CAD, compared to visual analysis of perfusion maps [4–6]. At present, only positron emission tomography (PET) imaging is capable of true perfusion quantification [7]. Morton et al. recently demonstrated potential for CMR to derive semiquantitative parameters of perfusion [8].

Before quantitative CT perfusion imaging can be implemented in clinical practice, systematic validation is mandatory. Therefore, an* ex vivo* model of a perfused, isolated heart of a slaughterhouse pig was used for this systematic analysis.

The aim of this study was to develop an explanted, perfused porcine heart model in a CT environment. We hypothesized that this model allows for standardized validation of CT perfusion parameters.

## 2. Materials and Methods

### 2.1. Heart Acquisition and Preparation

In this experiment, one* ex vivo* porcine heart model was used. The current study was designed to purely show the feasibility of the model. The heart was obtained from a pig, slaughtered for human consumption. Protocols at the slaughterhouse and laboratory were in accordance with EC regulations 1069/2009 regarding the use of slaughterhouse animal material for diagnosis and research, supervised by the Dutch Government (Dutch Ministry of Agriculture, Nature and Food Quality) and approved by the associated legal authorities of animal welfare (Food and Consumer Product Safety Authority).

A Dutch Landrace hybrid pig of approximately 110 kg live weight was used. After exsanguination, the thorax was opened by a parasternal incision. The pulmonary artery was cut just before the bifurcation. The aorta was cut before the supra-aortic vessels. The heart was immediately cooled topologically in ice slurry. To arrest the heart, the aorta was cannulated to administer 2 L of cold cardioplegic solution (4°C Custodiol histidine-tryptophan-ketoglutarate (HTK), Essential Pharmaceuticals, Pennsylvania, USA) to the coronary arteries at a pressure of 60 mmHg. The ischemic time at body temperature did not exceed 5 minutes. From subsequently slaughtered pigs, 20 L of fresh blood was collected for reperfusion. The heart and blood (heparinized with 5000 IU/L) were stored cold during transportation. Preparations of the heart were executed in the laboratory under cold and cardioplegic conditions until resuscitation. First, the pericardial sack was discarded. The azygos vein and the inferior and superior caval vein were ligated. A cannula was inserted into the aorta and fixed approximately 40 mm distal to the valve annulus. The pulmonary veins were cut at the left atrium and ligated. A perforated drain cannula was inserted into the left atrium and ventricle allowing sufficient venting after resuscitation. Similarly, a cannula was inserted into the pulmonary artery. Around the proximal circumflex (Cx) coronary artery, an inflatable cuff was placed, providing the possibility of mimicking stenosis under controlled circumstances. By measuring pressure distal to the stenosis and calculating the ratio between this pressure and the aortic pressure, the fractional flow reserve (FFR) was determined, a measure for the hemodynamic severity of the stenosis. In clinical practice, FFR measurements are performed under maximal hyperaemic conditions, using adenosine or dipyridamole. The heart in this experiment was already in maximal hyperaemic state, because of its removal from the pig body [9]. The Cx artery was chosen because it is easily accessible (in contrast to the right coronary artery) and perfuses an identifiable, but not too large, part of the left ventricle (in contrast to the left anterior descending artery). During the experiment, FFR measurements were used to verify the cuff-induced stenosis grade. CT perfusion scanning was first performed without stenosis, followed by scanning at FFR values of 0.7, 0.5, 0.3, and 0.1/0.0. Scanning was repeated three times at each FFR. A deviation of 0.05 from the goal FFR was considered acceptable. To prevent the contrast agent from building up in the circulating blood pool and thus causing differential baseline enhancement, the blood pool was refreshed with new blood after each stenosis grade situation.

### 2.2. Heart Perfusion

**Figure 1:**
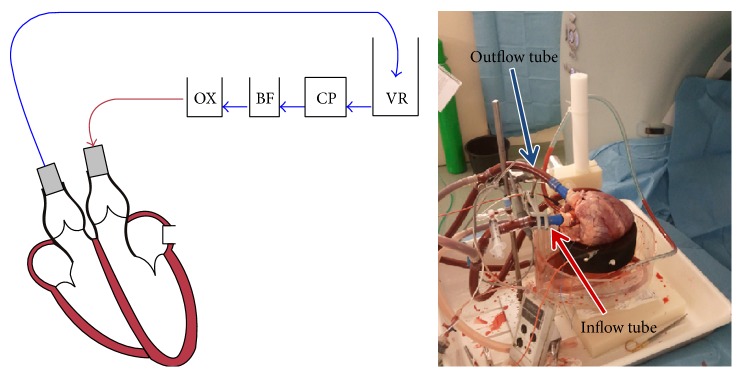
From the venous reservoir (VR), the blood first passed the cardiac pump CP. Then, the blood was pumped through the blood filter (BF) into the oxygenator (OX) and, from there, through the aorta into the coronary arteries. The setup was placed on the scanner table; all parts which could possibly interfere with the signal were placed outside the field of view.

After preparation, the aorta and pulmonary artery were connected to the circulation loop using the cannulas (Figure 1). A bed of flexible cloth provided epicardial suspension. The heart was aligned in the scanner in supine position. A modified Langendorff perfusion model was used, with an artificial heart-lung loop. In 1895, Langendorff et al. proposed a model of retrograde perfusion of mammalian hearts in which a Krebs-Henseleit solution circulated via the aorta [10, 11]. This Langendorff model was refined by circulation of whole blood [12]. Whole blood was pumped by a centrifugal pump (BioMedicus, Medtronic, Minneapolis, MN, USA) from a venous reservoir into the aorta towards the aortic sinus and the coronary arteries. The flow in the coronaries is pulsatile and pressure dependent. A pressure is present at the aortic root, causing the flow of blood into the coronaries. When the myocardium contracts during systole, the coronary vasculature is compressed and therefore the vascular resistance increases, resulting in reduced coronary flow. During diastole the myocardium relaxes, which opens the vascular bed and thereby lowers vascular resistance with higher coronary flow as a result. In a normally functioning heart, the aortic blood pressure pulse varies in pressure between 120 and 80 mmHg, resulting in a pressure pulse of 40 mmHg. However, in the Langendorff experiments the pressure pulse is only in the order of magnitude of 10 mmHg.

The coronary venous blood returned to the venous reservoir via the coronary sinus, right atrium, right ventricle, and pulmonary artery. The reperfusion medium circulated through a filter (AFFINITY Arterial 38 *μ*m blood filter; Medtronic, Minneapolis, Minnesota, USA) and an oxygenator-heat exchanger (AFFINITY NT Oxygenator; Medtronic, Minneapolis, Minnesota, USA). The blood was oxygenated with 20% O_2_, 75% N_2_, and 5% CO_2_ carbogen gas. Blood glucose level was maintained between 5 and 7 mmol/L by addition of glucose-insulin mixture. The temperature of the circulating blood was 38°C. The blood flow through the coronary arteries was approximately 1.5 mL/g per minute and controlled with the centrifugal pump. The mean coronary flow was measured with an ultrasound flow probe (LifeTec Group, Eindhoven, Netherlands) at the pump, and the pressure was measured at the aortic root with a pressure sensor (P10EZ-1; Becton Dickinson Medical, Franklin Lakes, New Jersey, USA).

When reperfusion of the coronary system was started, total ischemic time was approximately 4 hours. After coronary perfusion was reinstated, the heart showed spontaneous contractions while warming up by the circulating whole blood and gradually regained sinus rhythm. Some defibrillations were performed, of 10–30 Joules, to restore sinus rhythm. The heart was kept in this condition for at least 15 minutes, to allow stabilisation at the membranal level. If the heart rhythm did not stabilize, or if rhythm became irregular during the experiment, a Medtronic external pacemaker model 5375 (Medtronic, Minneapolis, Minnesota, USA) was used to induce a stable heart rate at approximately 70 beats per minute (see movie in Supplementary Material available online at http://dx.doi.org/10.1155/2015/412716). After obtaining a stable heart rate, the imaging protocols were started. ECG clips were placed on the “wet” flexible cloth and connected to the scanner ECG leads to feed signal to the scanner and allow ECG-synchronized image acquisition. During the entire experiment, the heart rate, pacing status (heart paced yes or no), aortic pressure, and blood flow were monitored. This enabled analysis of model stability and influence of luminal narrowing on the model.

### 2.3. CT Imaging Protocol

CT acquisitions were performed on a second-generation DSCT scanner (SOMATOM Definition Flash, Siemens Healthcare, Forchheim, Germany). Scans were acquired in caudocranial direction. At each stenosis grade, the protocol consisted of a noncontrast enhanced CT scan, three dynamic CT perfusion (CTP) acquisitions, and one coronary CT angiography (CTA) scan. First, scout images were obtained to determine the area of interest and a noncontrast enhanced CT scan was performed to determine baseline enhancement of the heart and blood pool. The unenhanced scan was made at tube voltage of 100 kV and tube current of 100 mAs. Next, perfusion series were performed at 5-minute intervals, to allow mixing of contrast agent in the blood pool and thus minimize the influence of contrast buildup. Dynamic perfusion image acquisitions were performed during end-systole (300 ms delay after the R-wave) in shuttle mode, with ECG-triggering. Given a detector width of 38.4 mm and an overlap of 10%, the anatomical coverage was 7.3 cm [13]. The scan area was determined based on the scout scan. The entire left ventricle of the heart was selected as image field of view (FOV), from the aortic root to the apex. The heart was placed parallel to the *z*-axis of the table. Matrix size was 512 × 512. Due to a heart frequency of >70 per minute, images were acquired every second heartbeat. Image acquisition parameters were 2 × 2 × 64 detector rows, 3.0 mm collimation, 2 × 100 kV tube voltage, and 350 mAs per rotation with a rotation time of 285 ms. The CM concentration was 300 mg iodine/mL (Ultravist 300, Bayer, Berlin, Germany), which was diluted to a mixture of 40 percent contrast and 60 percent saline for the injections. CM was injected into the blood stream 200 cm from the coronary arteries, allowing mixing of the perfusate and the CM but preserving the bolus shape of the injection. A CM volume of 15 mL, with a contrast to saline ratio of 40/60, was used at an injection rate of 3 mL/sec. Imaging was started 5 seconds prior to start of CM injection, with a total scan time of 35 seconds. After the perfusion series, coronary CTA scanning was performed. First, scan timing was determined by administering additional 10 mL of diluted (40/60 contrast/saline ratio) iodine contrast. Image acquisition was initiated 3 seconds after peak enhancement in the aortic root. Then, CTA was performed with another 15 mL of diluted contrast at an injection rate of 3 mL/sec. Coronary CTA data were acquired with retrospective ECG-gating, to analyse best-systolic and best-diastolic phase reconstructions. Scan parameters were 2 × 2 × 64 detectors rows, 0.6 mm detector collimation, 2 × 100 kV, 350 mAs per rotation, and 285 ms gantry rotation time. CTA data were acquired to analyse the stenosis severity related to the FFR measurements.

### 2.4. Image Reconstruction

Coronary CTA datasets were reconstructed with 0.75 mm slice thickness, 0.3 mm increment, and I26F (iterative) reconstruction kernel to reduce image noise. Coronary evaluation was performed in Syngo.via, based on curved multiplanar reformat series. The degree of luminal narrowing of the different stenosis grades was assessed by measuring the remaining luminal area on cross-sections. Mean diameter and area of the induced stenosis were compared to the FFR measurement to provide information on the severity of luminal narrowing. For myocardial evaluation, CT perfusion datasets were reconstructed in short-axis images with 3.0 mm slice thickness, 1.5 mm increment, and B23f (filtered back) reconstruction kernel for quantitative myocardial assessment, including an iodine beam hardening correction algorithm. Perfusion datasets were analysed using commercially available software, volume perfusion CT (VPCT) myocardium (VA41A, Siemens Healthcare, Forchheim, Germany). The AHA segmental model was used to label the segments of the heart [14]. The flow territory of the Cx was determined based on the total occlusion scan. Segments in the Cx territory were defined as Cx segments, others as non-Cx segments. Perfusion parameters for Cx myocardial segments and non-Cx segments were calculated. The FOV included the short-axis view of the heart. The inflow tube of the perfusate to the heart was looped through the FOV to allow calculation of an input function as reference for the perfusion of individual myocardial segments. The VPCT software uses the input function and the enhancement in the segments of the heart to calculate myocardial blood flow and blood volume in mL/100 mL/min and mL/100 mL, respectively, 100 mL being a measure for the volume of the myocardial tissue.

### 2.5. Statistical Analysis

Data management and statistical analysis were performed using Excel and SPSS 19 (IBM Corp, Armonk, NY). Independent variance tests were carried out to analyse whether the Cx and non-Cx segment groups showed normal distribution of perfusion measurements. Thereafter, the Mann-Whitney *U* test for equality was performed. Median values of myocardial blood flow and volume were compared between segments with normal perfusion and segments with induced stenosis at different stenosis grades.

## 3. Results

The pacemaker-induced heart rhythm was stable at approximately 70 beats per minute (supplementary movie). Arterial blood flow and blood pressure were kept constant as much as possible at 1 L/min and 80 mm/Hg, respectively. Blood flow was 1.0 L/min at FFR values of 1.0, 0.7, and 0.5 and slightly lower, 0.9 L/min, at FFRs of 0.3 and 0.1/0.0 (Table 1). Mean arterial pressure gradually increased from 80 to 95 mm/Hg over the course of the experiment. Stenosis induction with FFR pressures of 0.3 and 0.0 caused the arterial pressure to increase with 10 mm/Hg and blood flow to decrease with 0.1 L/min. The FFR measurements at each stenosis grade were stable. Occasionally, the pressure of the inflatable cuff lowered, recognisable as a rise in the FFR value. Then, the cuff was inflated again to maintain FFR within the 0.05 boundary from the goal stenosis grade. CTA measurements of the stenosis diameter and stenosis area are shown in Table 2. A 0.70 FFR-based stenosis corresponded with a 50 percent area stenosis on CTA.

**Figure 2:**
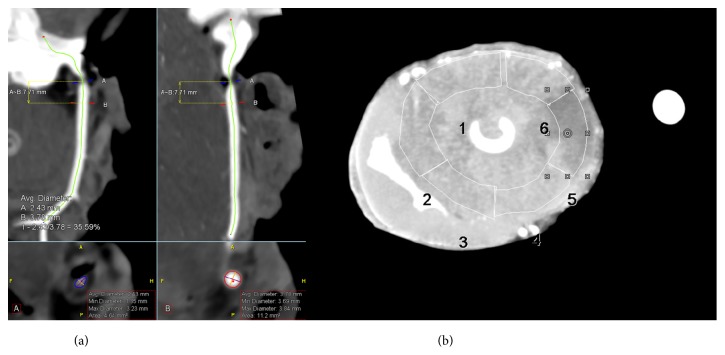
Example of images of the Cx artery with an FFR of 0.30. (a) The computed tomography angiography (CTA) image in curved multiplanar reformat shows the luminal narrowing. (b) The resulting perfusion defect in segment 5 as visible on maximum intensity projection (MIP) image.

**Figure 3:**
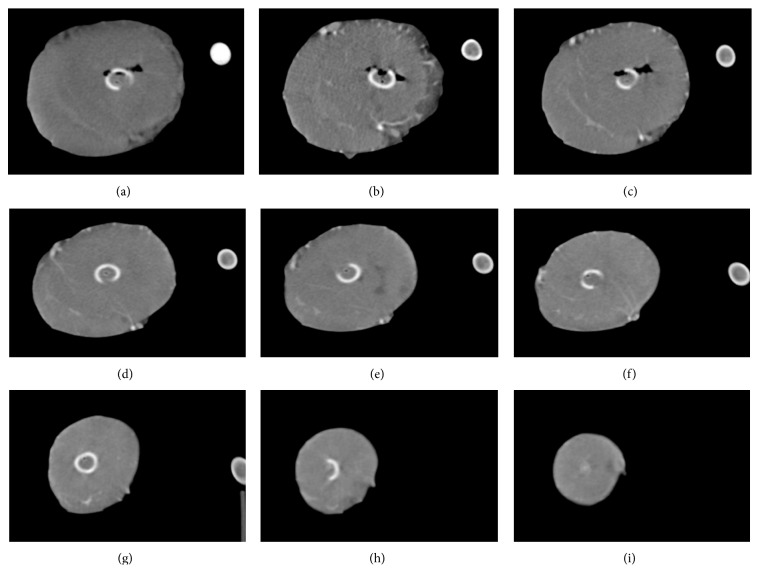
Image (a) shows the inflow of CM bolus through the inflow tube. Images (b)–(i) show the contrast inflow in the myocardium at short-axis cross-sections from basal to apical.

**Figure 4:**
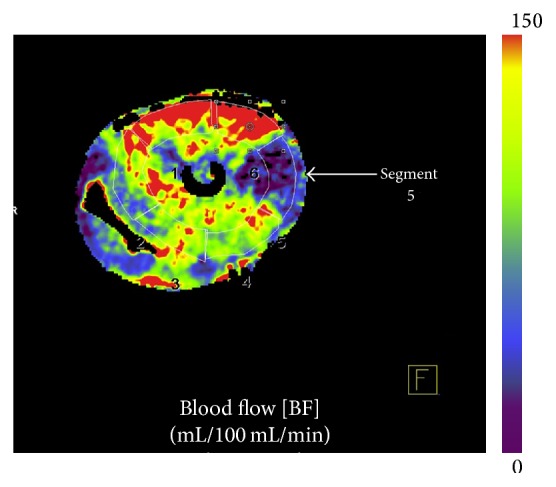
Blood flow map of the porcine heart at complete Cx occlusion: red color meaning higher/normal flow and blue indicating reduced flow.

Image quality of the coronary CTA scans was high, with HU values of over 325 in the coronary arteries (Figure 2). In the dynamic scans, the heart could be imaged in total (Figure 3). Based on the total occlusion scan, the flow territory of the Cx was found to be limited to segment 5 (Figures 2 and 4). The relatively limited extent of ischemia was due to a small size Cx artery in this porcine heart.

At every stenosis grade, three scans with one stenotic Cx segment and 15 normally perfused segments were analysed, a total of 48 segments per stenosis grade. In Table 3 the median values of myocardial blood flow and blood volume are shown for Cx- and non-Cx-perfused segments. Myocardial blood flow and volume for the Cx segment were lower at stenosis grades with FFR of ≤0.50 compared to non-Cx segments.

## 4. Discussion

This study shows the feasibility of myocardial perfusion analysis in a Langendorff pig heart model in a CT environment. This experimental setup enables detailed and systematic study of myocardial perfusion under standardized conditions and at differing degrees of blood flow, allowing for qualitative and quantitative evaluation of myocardial perfusion. Preliminary results suggest that newly developed CT perfusion imaging techniques can be validated with this model under controlled conditions, with hemodynamic settings relatively similar to the* in vivo*, clinical situation.

The Langendorff model is a retrograde perfusion model established in 1895 [11]. In 2007, Skrzypiec-Spring et al. reviewed the use of this model throughout a century of existence and concluded that it can be used to study ischaemia, stunning, hibernation, and arrhythmias and for drug testing [10]. In several studies the Langendorff model has been used to investigate, for instance, cardiac physiology and donor heart preservation methods [15, 16]. In our study, arterial blood flow and pressure were stable and controllable during most of our experiment. Part of the controllable circumstances included the confirmation of the stenosis degree by FFR assessment. The decrease in blood flow and increase in blood pressure during the experiment can be explained by the increase in peripheral resistance due to the induced stenosis. Because the heart was explanted, it was already in stress state and no compensatory mechanisms were present [9]. Thus, induction of a stenosis directly influenced coronary blood flow and pressure. A stenosis with a 0.7 pressure drop, however, did not influence blood flow and blood pressure in this single experiment. We hypothesize that the lumen area may still be too large for peripheral resistance to increase, and, therefore, blood flow and arterial pressure will not be influenced.

Schuster et al. showed potential for the Langendorff model for perfusion analysis in MRI environment [17]. They showed consistent blood flow and pressure over time for hearts without stenosis. However, they did not evaluate the effect of increasing coronary stenosis on model stability (RCA in one heart was occluded but only to compare first pass perfusion area to infarct size).

Small clinical studies have shown that adenosine stress dynamic perfusion CT testing can detect hemodynamically significant stenosis [3, 4, 18–20]. Quantitative evaluation of myocardial perfusion may have incremental value to detect flow-limiting stenosis compared to visual analysis. Prior to clinical implementation, the relationship between CT-derived perfusion parameters across the range of coronary stenosis needs validation against reference standards. Ethically, it is difficult to justify comparison of two modalities with associated radiation dose in patients. Also, there is little control over hemodynamic parameters in a human model especially regarding degree of stenosis. Conversely, a phantom model only allows limited conclusions with regard to the* in vivo* situation. An animal model mimicking human cardiac circulation may be of great help. Pigs have been researched extensively in cardiac imaging, since the porcine model provides excellent comparison to the human heart in size and physiology [21–24]. The porcine heart is especially well suited for myocardial perfusion imaging studies, because it cannot develop collateral flow. Thus, there is a direct relationship between stenosis grade and the decrease in downstream perfusion.

In* in vivo* porcine experiments CT allowed quantification of coronary flow [5, 22–25]. The advantage of* in vivo* experiments is that the physiology and cardiac function is intact providing cardiac features comparable to human studies. However,* in vivo* experiments are often more complex than single-heart experiments, because whole pigs cannot as easily and directly be manipulated as explanted hearts. Physiologically, the explanted heart is not as complex as the heart in living animals, offering more control and possibly better reproducibility. An extension of the present experiment could lie in imaging the porcine heart with different modalities to directly compare perfusion techniques at different stenosis grades.

Summarizing, our model has several benefits compared to other experimental models. A major advantage of the Langendorff model is the control of myocardial blood flow. A stenosis can be directly induced, and its severity can easily be altered. Because the heart is disconnected from the body, it is free of neural/hormonal influences. The heart is already in stress state, because of the prior explantation. Thus, the coronary arteries are at maximum dilation, mimicking the situation of adenosine stress perfusion imaging. Furthermore, after otherwise fatal events such as arrhythmias and infarction-induced cardiac arrest, introducing a pacemaker or reinstating sinus rhythm with defibrillations of 10–30 J can prolong the experiment. Compared to scanning and subsequent sacrificing of living pigs, a model using slaughterhouse pigs provides an alternative where no additional animals are sacrificed.

Our model also has some disadvantages. Due to the fact that physiological processes were no longer intact, the model is less comparable to the clinical situation. The setup platform and fixation as well as contrast protocol and left ventricular pressure are not like* in vivo* situations. The setup was mostly made of plastic, because metal would induce large artifacts. Furthermore, the heart experienced an ischaemic period directly after removal, which may alter its condition and influence perfusion. During the experiment, some difficulties in ECG-triggering were experienced. This could be due to the usage of the pacemaker. The signal transduction of the pacemaker spike within the heart can differ between beats, possibly resulting in phase irregularities. Even so, signal transduction during the experiment was generally good, with good image quality and limited motion artifacts. Pacemaker-induced rhythm is generally stable; however premature ventricular contractions may occur. Another possible bias is the fixed pacemaker-induced heart rate in this heart. Previous studies in humans have shown an increase in heart rate when stressed using adenosine [18, 19]. This increase is not simulated in this particular pig heart experiment. Therefore, our Langendorff experiment could be less prone to heart rate dependent artefacts compared to human studies when heart rates are higher. The stenosis induced in the Cx artery did not cause a large perfusion defect, because of a rudimentary Cx artery. There is a natural distribution in the size and perfusion areas of the coronary arteries in pigs, comparable to humans. We intend to repeat this experiment in a number of pig hearts to gain more information on variability of the model. Compared to an entire pig, the current model is relatively expensive. A last limitation is that the study was performed in one pig heart, only. The current study was designed to purely show the feasibility of the model. The model should be repeated in a larger number of explanted hearts to confirm stability of the setup and to optimize the experimental protocol.

## 5. Conclusion

This study demonstrates the feasibility of* ex vivo *myocardial perfusion imaging and quantification in a CT environment at different grades of coronary stenosis. The Langendorff model provides control over physiological parameters such as blood flow and stenosis grade. The described model shows promise for validation of CT perfusion imaging techniques under controlled circumstances.

## Supplementary Material

Supplementary movie shows the working model of the ex-vivo pig heart with perfusion according to Langendorff.

## Figures and Tables

**Table 1:** Arterial blood flow (L/min) and blood pressure (mm/Hg) in 5 different stenosis grades.

	Normal perfusion	FFR 0.70	FFR 0.50	FFR 0.30	Total occlusion
Arterial blood flow (SD)	1.0 L/min (constant)	1.0 L/min (constant)	1.0 L/min (constant)	0.93 L/min (SD 0.05)	0.90 L/min (constant)

Arterial blood pressure (SD)	82.7 mm/Hg (SD 0.6)	80.7 mm/Hg (SD 1.2)	86.7 mm/Hg (SD 1.2)	88.7 mm/Hg (SD 1.5)	94 mm/Hg (SD 2.7)

SD: standard deviation.

**Table 2:** Diameter of stenosis divided by the artery diameter before stenosis, and the area stenosis divided by the area before stenosis, for the FFR-based stenosis grades.

	FFR 0.70	FFR 0.50	FFR 0.30
Mean diameter on CTA	74%	47%	31%
Area stenosis on CTA	49%	22%	12%

**Table 3:** Median blood flow (L/min) in normal and defected segments for multiple stenosis grades.

	No stenosis	FFR 0.70	FFR 0.50	FFR 0.30	Total occlusion
Calculated normal segments (min–max)	151 mL/100 mL/min (113–205)	173 mL/100 mL/min (122–260)	162 mL/100 mL/min (133–247)	124 mL/100 mL/min (70–207)	108 mL/100 mL/min (43–167)

Calculated defect segments (min–max)	133 mL/100 mL/min (127–139)	157 mL/100 mL/min (129–183)	121 mL/100 mL/min (96–125)	74 mL/100 mL/min (66–78)	34 mL/100 mL/min (34–39)

Minimum and maximum blood flow for each group are shown in brackets.

## Abbreviations

CAD: Coronary artery disease
CM: Contrast media
CT: Computed tomography
CTA: Computed tomography angiography
CTP: Computed tomography perfusion
Cx: Circumflex artery
FOV: Field of view
FFR: Fractional flow reserve
MRI: Magnetic resonance imaging
DSCT: Dual source computed tomography
PET: Positron emission tomography
RCA: Right coronary artery
VPCT: Volume perfusion CT.
